# Hydrogen Peroxide-Induced Senescence Reduces the Wound Healing-Promoting Effects of Mesenchymal Stem Cell-Derived Exosomes Partially via miR-146a

**DOI:** 10.14336/AD.2020.0624

**Published:** 2021-02-01

**Authors:** Meiqian Xu, Xiaodong Su, Xian Xiao, Hongliang Yu, Xiaoxia Li, Armand Keating, Shihua Wang, Robert Chunhua Zhao

**Affiliations:** ^1^Institute of Basic Medical Sciences Chinese Academy of Medical Sciences,School of Basic Medicine Peking Union Medical College, Center of Excellence in Tissue Engineering Chinese Academy of Medical Sciences, Beijing 100005, China.; ^2^Brain Tumor Research Center, Beijing Neurosurgical Institute, Beijing Tiantan Hospital Affiliated to Capital Medical University, Beijing Laboratory of Biomedical Materials, Beijing 100070, China.; ^3^Department of Genetics and Cell Biology, Basic Medical College, Qingdao University, Qingdao 266071, China.; ^4^Cell Therapy Translational Research Laboratory, Princess Margaret, Cancer Centre, Toronto, Ontario M5G 2M9, Canada.; ^5^Institute of Biomaterials and Biomedical Engineering, University of Toronto, Toronto, Ontario M5G2M9, Canada.; ^6^Institute of Medical Science, University of Toronto, Toronto, Ontario M5G 2M9, Canada.

**Keywords:** MSC, exosome, miR-146a, angiogenesis, senescence

## Abstract

Mesenchymal stem cells (MSCs) have beneficial effects on wound healing. MSCs function through direct cell-cell communication or indirectly through paracrine secretion of exosomes. Here, we found that MSC-derived exosomes had pro-wound healing effects via promotion of angiogenesis; however, this promoting effect was significantly reduced when senescence was induced in parental MSCs by hydrogen peroxide (H_2_O_2_). Further experiments showed that decreased miR-146a expression in exosomes derived from senescent MSCs (s-exo) contributed to these findings. *In vitro*, the pro-angiogenic effect of s-exo on tube formation in human umbilical vein endothelial cells was significantly reduced compared with that of exosomes derived from control MSCs (c-exo). *In vivo*, higher tube numbers and longer tube lengths were observed in the c-exo group compared with the s-exo group. Using microarray analysis, we found that miR-146a level in s-exo was lower than that in c-exo. Knockdown of miR-146a in c-exo decreased its capacity to promote angiogenesis, and overexpression of miR-146a in s-exo partially rescued its impaired pro-angiogenic capacity, thereby confirming that downregulation of miR-146a contributed to the reduced pro-wound healing capacity of s-exo. Our study is the first to demonstrate that cell senescence induced by H_2_O_2_ alters the pro-angiogenic ability of exosomes by modulating the expression of exosomal miRNAs, especially miR-146a, thus providing new insights into the correlation between parental cell state and exosome content and function.

Skin injuries are the most common type of accidental injuries. Skin wound healing is a complex and coordinated process that involves cell proliferation and migration, extracellular matrix synthesis and deposition, angiogenesis, and remodeling [1] . Although a normal wound can heal by itself, there are certain disorders, such as diabetes, which can disturb the healing process, leading to non-healing wounds [2]. Accelerating wound healing can promote restoration of physiological homeostasis in the damaged tissues. Conventional interventions for inadequate wound healing include mechanical debridement and dressing changes. However, in several circumstances, these methods do not provide satisfactory results. For example, aging skin is associated with an increased risk of chronic non-healing wounds [3], and diabetic patients often develop painful wounds that may never heal[4] . Therefore, exploring novel treatments to promote wound healing has become the focus of regenerative medicine. Currently, a potential therapeutic strategy using exosomes has gained much attention.

Exosomes are cup-shaped nanoparticles (30-150 nm) with a lipid bilayer membrane [5].They are secreted by various cell types and are present in all kinds of body fluids, such as serum [6], saliva [7], urine [8], and milk [9]. As an important component of paracrine secretion, exosomes can transfer bioactive molecules, such as proteins, DNA, lipids, non-coding RNAs, mRNAs, and microRNAs (miRNAs) into target cells, facilitate cell-to-cell communication, and induce functional changes [10]. Exosomes have the capacity to protect cellular contents from enzymatic or chemical degradation [11]. Studies have shown that exosomes can improve wound healing and tissue regeneration [12]. As the healing process is complicated, exosomes may affect multiple steps of the process. Moreover, exosomes have been shown to exhibit angiogenic function. Yin *et al*. [13]demonstrated that exosomes isolated from human umbilical cord blood induced promotion of angiogenesis and fibroblast function. Prabhu *et al*. [14] showed that human CD34^+^ stem cell-derived exosomes could repair ischemic hindlimb through angiogenic mechanisms. Chen *et al.* [15] found that exosomal factors facilitate diabetic wound repair by promoting angiogenesis. Furthermore, it has been reported that exosomes can accelerate wound closure through facilitating proliferation of epithelial cells and altering the characteristics of fibroblasts. Shang-Chun Guo *et al*. [16]showed that platelet-rich exosomes promoted re-epithelization. Li Hu *et al*. [17] revealed that exosomes can accelerate cutaneous wound healing via optimizing the characteristics of fibroblasts. Since exosomes may represent a promising non cell-based biotherapy for wound healing, it is important to determine the optimal parental cell state in order to achieve maximal therapeutic effects. Considering that exosomes are a kind of secretory component, it is reasonable to hypothesize that their function is largely depended on the physiological state of their parental cells. It has been reported that hypoxia exposure can enhance the angiogenic effect of mesenchymal stem cell (MSC)-derived exosomes [18], and autophagy can inhibit exosome release [19]. However, whether aging affects exosome function remains largely unknown. As MSC-derived exosomes is one of the most frequently used molecules in exosomal therapy, an acute injury-induced senescence would lead to fundamental changes in the cells. In this study, we used exosomes derived from control MSCs (c-exo) or hydrogen peroxide (H_2_O_2_)-induced senescent MSCs (s-exo) to explore the changes in exosomal contents and functions with cell state and the underlying molecular mechanism.

No statistically significant difference in morphology, marker expression, and quantity was found between the two exosome types. Human umbilical vein endothelial cells (HUVECs) could actively and indistinguishably take up c-exo and s-exo. However, the wound healing-promoting effect of exosomes was largely reduced when parental cells were senescent. Further experiments demonstrated that exosomes exhibited pro-angiogenic capacity *in vitro* and *in vivo* and they promoted wound healing through facilitating angiogenesis. Mechanistically, we found that parental cell senescence caused significant transcriptomic alterations in exosomes and chose miR-146a as the candidate molecule. To confirm the exosomal function of miR-146a, c-exo and s-exo with inhibition and overexpression of miR-146a were obtained, respectively. Results revealed that downregulation of miR-146a expression in c-exo partially inhibited its pro-angiogenic effect and upregulation of miR-146a in s-exo partly rescued the reduced promoting effect. Additionally, our findings showed that the pro-angiogenic capacity of exosomes derived from MSCs was reduced when the parental cells were senescent, and this decrease depended on the expression changes of exosomal miRNAs, including miR-146a. Hence, our study provides new insights into understanding the correlation between parental cell state and exosome function and developing optimization strategies to isolate functional exosomes from MSCs.

## MATERIALS AND METHODS

### Cell culture

Human adipose tissues and umbilical cords were obtained according to the procedures approved by the Ethics Committee at the Chinese Academy of Medical Sciences and Peking Union Medical College. MSCs were isolated and culture-expanded from healthy volunteers as previously described [20]. MSCs were resuspended in 12 mL culture medium and seeded at a density of 1.7 × 10^5^ cells/ml in a 75 cm^2^ culture flask. Cells were maintained at 37 °C in a humidified incubator with 5% CO_2_ and passaged with trypsin/EDTA after reaching confluence. Passage 3 cells were used for all subsequent experiments. Human umbilical vein endothelial cells (HUVECs) were isolated and cultured in endothelial cell medium containing endothelial cell growth basal medium, endothelial cell growth supplement, and 5% fetal bovine serum (ECM #1001; ScienCell), as previously described [21].

### Induction of cell senescence

To induce cell senescence, MSCs were subjected to oxidative stress with H_2_O_2_. Cells at half-confluence were exposed to various doses of H_2_O_2_ (0, 50, 100, 200, and 400 μM) for 2 h. Following this, cells were washed with phosphate-buffered saline (PBS) and cultured in fresh media for 3 days. To evaluate cell senescence, the β-galactosidase assay was performed using the senescence β-galactosidase staining kit (Beyotime) according to the manufacturer’s instructions.

### Exosome isolation

Exosome extraction was performed as previously described. Briefly [22], MSCs were cultured in serum-free DMEM/F12 medium for 48 h. The culture medium was then collected and centrifuged at 800 *g* for 5 min and an additional 2000 *g* for 10 min to remove lifted cells. The supernatant was subjected to filtration on a 0.1 mm pore polyethersulfone membrane filter (Corning) to remove cell debris and large vesicles, followed by concentration with a 100,000-Mw cutoff membrane (CentriPlus-70; Millipore). The volume of the supernatant was reduced from approximately 250-500 mL to less than 5 mL. The supernatant was then ultracentrifuged at 100,000 *g* for 1 h at 4 °C using the 70Ti rotor (Beckman Coulter). The resulting pellets were resuspended in 6 mL PBS and ultracentrifuged at 100,000 *g* for 1 h at 4 °C using the 100Ti rotor (Beckman Coulter).

### Transmission electron microscopy

Purified exosomes were fixed in 1% glutaraldehyde in PBS (pH 7.4). After rinsing, a 20 μL drop of the suspension was loaded onto a formvar/carbon-coated grid, negatively stained with 3% (w/v) aqueous phosphotungstic acid for 1 min and observed under a transmission electron microscope.

### Nanoparticle tracking analysis

Exosome pellets were resuspended in PBS. Exosome sizes were measured by nanoparticle tracking analysis with ZetaView (Particle Metrix), according to the manufacturer’s instructions. Usually, the exosomes are diluted 100-400 times in 100 μL of sterile PBS before analysis.

### Western blotting

Proteins were extracted with radioimmunoprecipitation (RIPA) lysis buffer with PMSF and quantified using a BCA protein assay kit (Beyotime). Western blotting was performed in triplicates according to a previously described protocol [23]. The following exosomal protein antibodies were used: CD63 (1:500, rabbit IgG; Proteintech, 25682-1-AP), HSP90 (1:1000, rabbit IgG; Proteintech, 13171-1-AP), calnexin (1:2000, rabbit IgG; Cell Signaling Technology, 2433s), horseradish peroxidase (HRP)-conjugated anti-rabbit-IgG, and HRP-conjugated anti-mouse-IgG (NeoBioscience).

### Exosome uptake assay

Exosome uptake was assessed by labeling the exosomes with 3,3-dioctadecyloxacarbocyanine perchlorate (DIO; 1:2000; Invitrogen) and co-culturing with HUVECs for 3, 6, 9, and 12 h. Excessive exosomes were then washed with culture medium, and the cell nuclei were stained with Hoechst 33342 (1:500; Sigma-Aldrich). Dye transfer was visualized by fluorescence microscopy (Olympus).

### Immunofluorescence staining

The cultured cells were fixed in ice-cold methanol at 4 °C for 10 min, washed three times with PBS, and then permeabilized in 0.1% Triton X-100/PBS for 10 min at room temperature. Nonspecific binding was blocked with 0.5% Tween-20/PBS containing 1% bovine serum albumin for 30 min. Cells were then incubated with primary antibodies at 4 °C overnight, followed by incubation with secondary antibodies were incubated for 1 h at room temperature. Next, cells were washed with PBS and stained with Hoechst 33342 (Sigma-Aldrich) for visualization of the nuclei. CD31 antibody was purchased from Proteintech (11265-1-AP; 1:100, mouse IgG).

### Quantitative real-time polymerase chain reaction (PCR)

Total RNA was extracted using TRIzol reagent (Invitrogen) according to the manufacturer’s instructions, followed by cDNA synthesis. Real-time PCR amplification was performed in triplicates according to a previously described procedure [24]. Relative mRNA expression was evaluated by the 2^-ΔΔCt^ method and normalized to GAPDH expression.

### Tube formation assay

*In vitro* capillary network formation was analyzed by the tube formation assay using Matrigel (BD Biosciences). HUVECs (1 × 10^4^/wells) were plated on a growth factor reduced Matrigel-coated 96-well plate in triplicates in 100 μL endothelial cell medium (control), endothelial cell medium with c-exo (10 μg), or endothelial cell medium with s-exo (10 μg). After 8 h of incubation, tube formation was examined by microscopy (Olympus) and the tube length was measured in three randomly selected fields per well.

### Mouse skin wound model

Female C57BL/6 mice (6 weeks old) were purchased from the Laboratory Animal Center of the Chinese Academy of Medical Sciences (Beijing, China). Animal use and experimental procedures were approved by the Animal Care and Use Committee of the Chinese Academy of Medical Sciences. The mice were kept under observation for one week before experimental procedures. The mice were anesthetized by intraperitoneal injection of 50 mg/kg pentobarbital sodium (Sigma-Aldrich) and a full-thickness excisional skin wound (6 mm in diameter) was made on the back of each mouse after shaving. The mice were randomly divided into three groups (n=5): PBS group (treated with 100 μL PBS), c-exo group (treated with 100 μg c-exo in 100 μL PBS), and s-exo group (treated with 100 μg s-exo in 100 μL PBS). Briefly, they were subcutaneously injected with c-exo, s-exo, or PBS around the wounds at 4 injection sites (25 μL per site). At days 0, 6, 9, and 12 post-wounding, wounds were photographed and measured. Wound-size reduction was calculated: wound-size reduction (%) = (A0 - At)/A0 × 100, where A0 is the initial wound area, and At is the wound area at the indicated times. At 12 days post operation, mice were sacrificed, and skin samples were harvested and fixed in 10% paraformaldehyde (PFA). Samples from each group were subjected to hematoxylin-eosin (H&E) staining for the detection of newly formed blood vessels.

**Figure 1.**
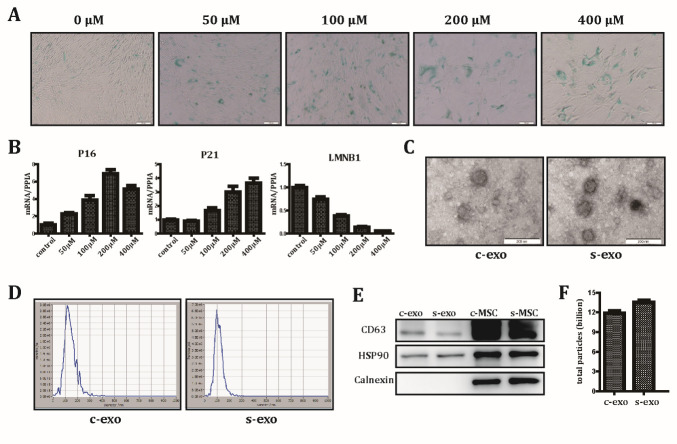
Characterization of exosomes derived from control MSCs and senescent MSCs. (A) Representative images of SA-β-gal staining with increasing concentrations of H_2_O_2_. Scale bar: 100 μm. (B) Enhanced expression of the senescence markers, P16 and P21, and reduced expression of LMNB1, a negative marker of aging, as detected by qRT-PCR. (C) Transmission electron microscopy of exosomes isolated from control MSCs (c-exo) and senescent MSCs (s-exo). (D) Nanoparticle size distribution of c-exo and s-exo. Scale bar: 200 μm. (E) The expression of exosome markers, CD63 and HSP90, and the cell-specific marker calnexin was analyzed by western blotting in exosomes and their producer cells. (F) The total secreted exosome particles collected from 1×10^8^ control MSCs (c-MSC) and senescent MSCs (s-MSC) grown for 48 h in FBS-free culture.

**Figure 2.**
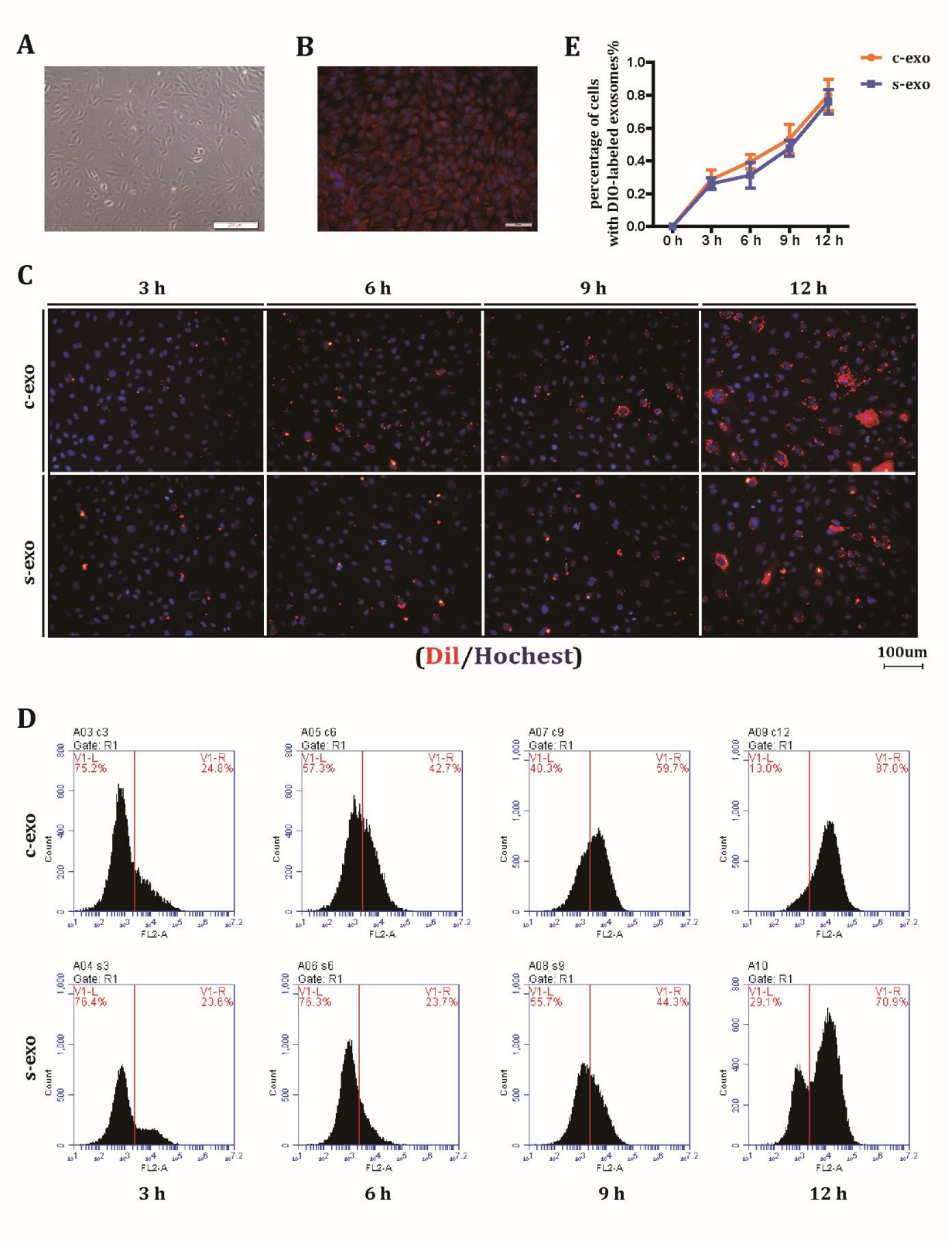
HUVECs can indistinguishably take up c-exo and s-exo. (A) Typical morphology of HUVECs. (B) Immunofluorescence staining of the endothelial marker CD31. (C) Uptake of c-exo and s-exo by HUVECs at 3, 6, 9, and 12 h after co-culture. (D) Quantification of c-exo and s-exo uptake by HUVECs at 3, 6, 9, and 12 h after co-culture through flow cytometry. (E) Quantitative flow cytometry results, as indicated by a line chart.

### Matrigel plug assay

Aliquots (150 μL) of growth factor reduced Matrigel (BD Biosciences) containing 5 × 10^6^ HUVECs and 150 μL of PBS, c-exo (100 μg), or s-exo (100 μg) were prepared on ice. Mice were randomly divided into three groups (PBS, c-exo, and s-exo) and anesthetized by intraperitoneal injection of 50 mg/kg pentobarbital sodium (Sigma-Aldrich). Matrigel aliquots were injected bilaterally into the inguinal areas and allowed to gel at body temperature. After 10 days, mice were euthanized for plug excision. Plugs were fixed in 4% PFA and subjected to CD31 staining for the detection of angiogenesis.

### Immunofluorescence histochemistry

Briefly, plug samples at day 10 post injection were fixed in 4% PFA, dehydrated in 30% sucrose solution, and embedded in OCT. Samples were then sectioned (10 μm thick sections) and incubated with CD31 antibody (1:100, mouse IgG; Proteintech; 11265-1-AP) overnight at 4 °C, followed by incubation with Cy3-conjugated secondary antibody (Abcam; ab97075; 1:200) at room temperature for 1 h away from light. Cells were then visualized and imaged using a fluorescence microscope.

### Transfection of cells and exosomes with miRNA mimics

Cells were seeded in a 6-well plate at a density of 2 × 10^5^ cells/well. After reaching 70% confluency, cells were transfected with miRNA mimics (GenePharma, Shanghai, China) according to the manufacturer’s instructions. After 6 h, the medium was replaced with fresh medium to minimize toxicity.

For exosome transfection, purified exosomes were transfected with miRNA mimics using Exo-Fect (System Biosciences) and incubated on ice for 30 min. Next, TC reagent (System Biosciences) was added to stop the reaction. The mixtures were then centrifuged at 13,000 rpm for 4 min to obtain exosomes with transfected miRNAs.

### Statistical analysis

Data are presented as mean ± standard deviation (SD). Comparisons between groups were made by Student’s *t*-test. Differences were considered statistically significant at *P < 0.05, **P < 0.01, and ***P < 0.001.

## RESULTS

### Characterization of c-exo and s-exo

H_2_O_2_ can induce oxidative stress injury and is frequently used to establish an aging or apoptotic model [25, 26]. To explore the optimum H_2_O_2_ modeling concentration for MSCs, cells were treated with increasing concentrations of H_2_O_2_. Senescence-associated β-galactosidase (SA-β-gal) staining was then performed to detect senescent cells. We found that cells treated with higher concentrations of H_2_O_2_ showed a higher percentage of SA-β-gal-positive cells (Fig. 1A). As shown in Figure 1B, the expression level of the senescence markers, p16 and p21, was significantly increased, while that of the negative marker LMNB1 was significantly downregulated in the H_2_O_2_-treated group compared with that in the control group, thereby indicating that oxidative stress injury induced by H_2_O_2_ drives cells into a senescent state. Since cell viability was not impaired and a senescent phenotype was detected at a concentration of 200 μmol, this concentration was selected for further experiments. To characterize the isolated exosomes, transmission electron microscopy and nanotracking analysis (NTA) were used to evaluate morphology and diameter distribution. Both groups of exosomes presented a typical cup-shaped appearance (Fig. 1C). The peak diameters of c-exo and s-exo were 106 and 130 nm, respectively, which is in accordance with the accepted exosome size range (30-150 nm) (Fig. 1D). No obvious size and morphology differences were observed between these exosome groups. Western blotting demonstrated enrichment of exosomal marker proteins, HSP90 and CD63, but absence of cell-specific protein calnexin in both c-exo and s-exo groups (Fig. 1E). To explore whether cell state has an impact on exosome secretion, the number of exosomes derived from the same volume of control and senescent MSCs was counted, and results showed no significant difference (Fig. 1F). Collectively, these data demonstrated that there was no difference in characteristics between c-exo and s-exo.

### HUVECs can actively take up c-exo and s-exo

Although c-exo and s-exo have the same characterization, whether can they be internalized indistinguishably by target cells is not well studied. We selected HUVECs as a model to assess exosome internalization because previous research reported that HUVECs can actively take up MSC-derived exosomes [27]. Primary HUVECs were extracted from the human umbilical vein. Positive expression of the endothelial marker CD31 (positive percentage >95%) and a typical cobblestone-shaped appearance confirmed cell identity (Fig. 2A and B). DIO-labeled c-exo and s-exo were then added to the culture medium, and exosome uptake was assessed by fluorescence microscopy at different time points after completely washing the cells to get rid of extracellular exosomes (Fig. 2C). The results showed that HUVECs began to internalize exosomes when co-incubated for 3 h, and exosome uptake reached a peak at 12 h. To quantify the percentage of cells with DIO-labeled exosomes, flow cytometry was performed to detect the number of positive cells (Fig. 2D). As indicated by the line chart, no significant differences were observed during the uptake process (Fig. 2E). These data suggested that both types of exosomes were indistinguishably internalized into the intracellular region.

**Figure 3.**
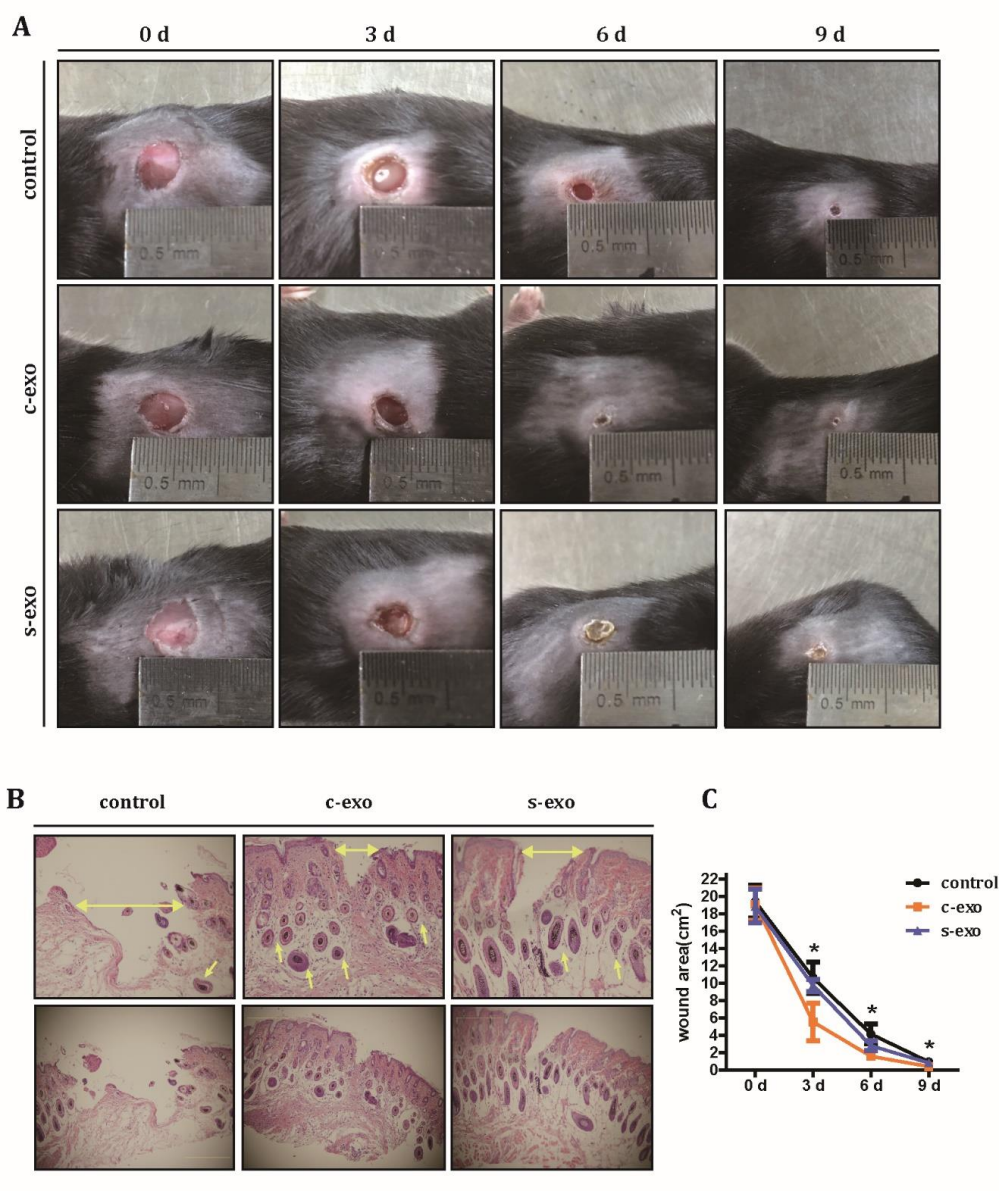
S-exo have reduced wound healing-promoting effect. (A) Representative cutaneous wound photographs at days 0, 3, 6, and 9 post treatment with PBS, 100 μg c-exo, and 100 μg s-exo. (B) H&E staining of the skin tissue around the wound at day 14 after operation (horizontal arrows indicated the wound width, and the other arrows indicate tubes around the wounds). Photos in top panel is the partial enlarged view of the bottom panel. (C) Quantification of the wound areas during the healing process. Asterisks indicates a significant difference between the c-exo and s-exo groups.

**Figure 4.**
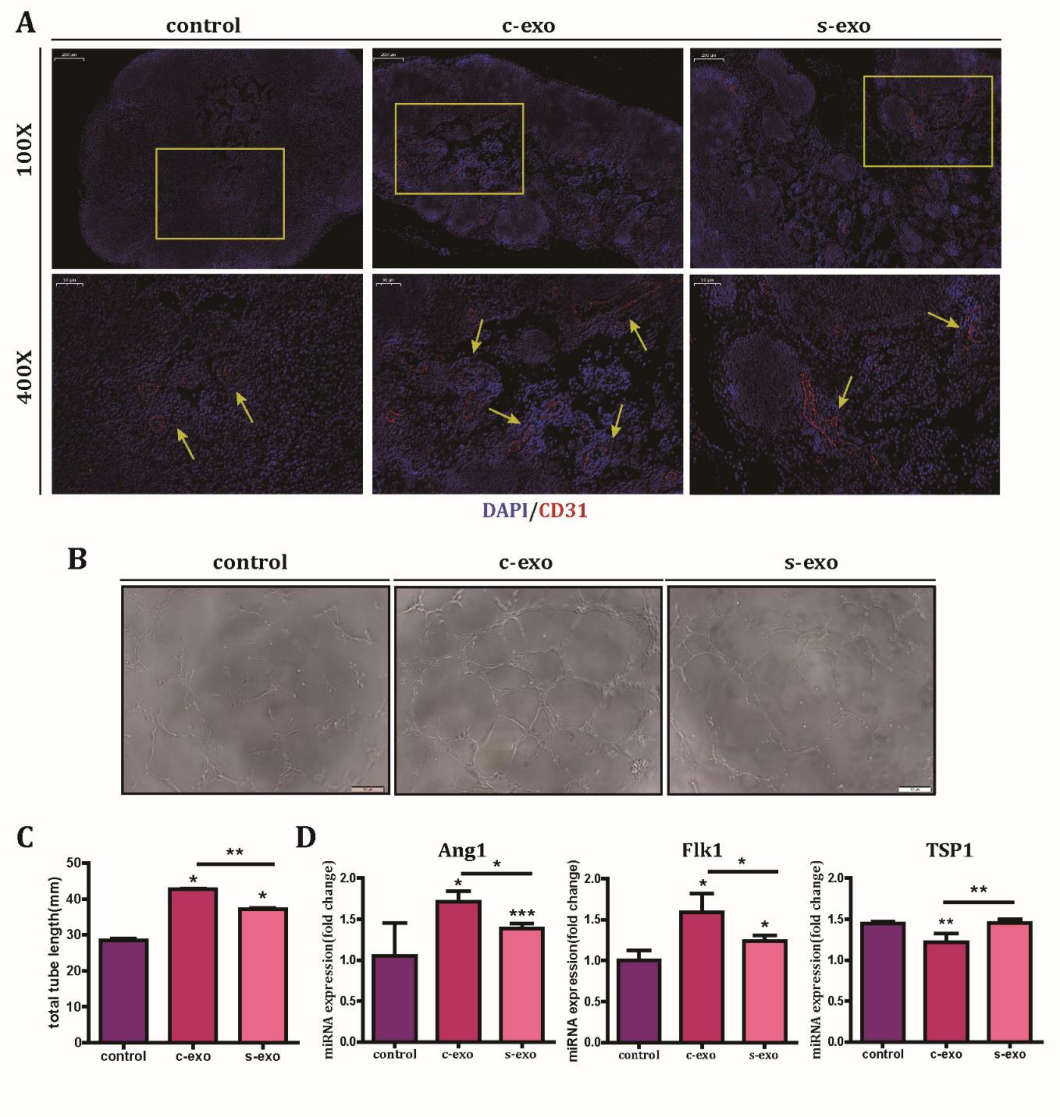
Senescent exosomes have reduced angiogenesis-promoting effect both *in vivo* and *in vitro*. (A) Representative images of immunofluorescence staining for CD31 in paraffin-embedded sections of Matrigel plugs (red staining). Arrows indicate positive staining for CD31. For Matrigel plug assay, Matrigel and HUVECs were mixed with c-exo, s-exo (100 μg exosomes; 2 × 10^7^ HUVECs per 200 mL Matrigel), or PBS and subcutaneously injected into C57BL/6mice. Matrigel plugs were obtained 10 days later. Scale bar: 200 μm (top panel) and 50 μm (low panel). (B) Tube formation in HUVECs cultured with PBS, c-exo, and s-exo was observed after 8 h of co-culture. (C) Quantification of tube length. (D) Quantification of angiogenesis-associated genes in HUVECs cultured with PBS, c-exo, and s-exo.

### The pro-wound healing effect of exosomes is reduced when parental MSCs are senescent

Next, we explored whether there are any functional differences between c-exo and s-exo. Since previous researches have shown that many types of MSC-derived exosomes possess wound healing capacity [28], we wanted to test the hypothesis that exosomes isolated from adipose-derived stem cells can promote wound healing, and when cell senescence occurs, the promoting effect may reduce or disappear. Cutaneous wounds were created on the backs of C57BL/6 mice to establish a skin injury model. After subcutaneous injection of c-exo, s-exo, or PBS around the wound, the healing process was analyzed at days 3, 6, and 9 post-wounding (Fig. 3A). Digital photographs of the wounds revealed that the percentage of wound closure in mice exposed to c-exo reached 91.84 ± 0.48% at day 6. However, mice treated with PBS or s-exo displayed only 77.32 ± 2.40% and 85.20 ± 1.20% wound closure, respectively. For better visualization of the wounds, H&E staining was performed at day 14 post-wounding. The results confirmed that both c-exo and s-exo groups had smaller wound sizes (174.54 ± 31.27 pixels and 288.62 ± 31.25 pixels) than the control group (758.47 ± 300.21 pixels). Interestingly, more tube formation was observed around the scar in both exosome groups compared with the control group (Fig. 3B). The result was confirmed immunocytochemical staining and ERG in skin samples (Supplementary Fig. 1). A complete wound area analysis was carried out during the entire healing process. As shown in Figure 3C, the wound area of the c-exo group was significantly smaller than that of s-exo group at each time point. In conclusion, these results indicated that exosomes derived from MSCs have wound healing-promoting effects, which are reduced when the parental cells are senescent.

**Figure 5.**
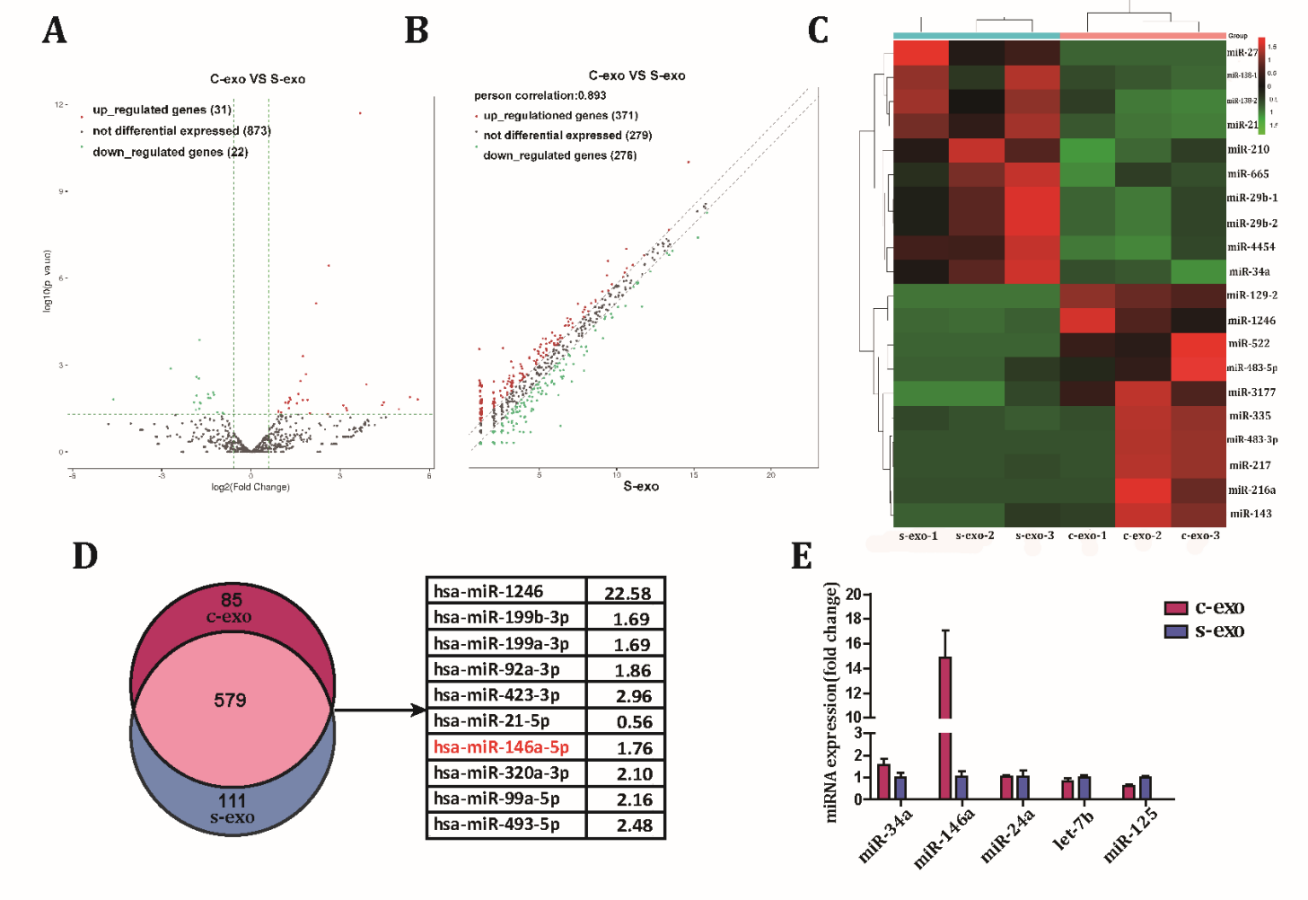
Different miRNA expression patterns between c-exo and s-exo. (A) A volcano plot of miRNAs expressed in c-exo and s-exo. (B) A scatter plot of differences in miRNA expression between c-exo and s-exo. (C) The top 10 up- and down-regulated miRNAs in c-exo and s-exo. (D) A Venn diagram showing the number of miRNAs identified in c-exo and s-exo. Of the 579 miRNAs commonly expressed by both c-exo and s-exo, the top 10 up-regulated miRNAs in c-exo compared with s-exo were selected (as indicated in the table). (E) Selected miRNAs associated with angiogenesis were analyzed by Q-PCR.

### The wound healing ability of s-exo is decreased due to its reduced pro-angiogenic capacity

Since wound healing is a complicated process, there are many functional factors involved in recovery. Angiogenesis plays an important role in the healing process, and previous results on tube formation around the scars indicated that exosomes may function through enhancing angiogenesis. To verify this assumption, tube formation assay was performed *in vivo* and *in vitro*. *In vivo*, before subcutaneous injection in the axilla, c-exo, s-exo, or PBS were mixed with HUVECs in growth factor-reduced matrigel. Immunohistochemistry results revealed that tube formation (indicated by CD31^+^ cells) was rarely observed in the PBS group, but appeared in the c-exo and s-exo groups (Fig. 4A). In accordance with the wound healing results, the angiogenic ability of the c-exo group was higher than that of the s-exo group. *In vitro*, we added c-exo, s-exo, or PBS into the culture medium of HUVECs. Microscopy photographs revealed a significant enhancement of tube formation in HUVECs incubated with c-exo (Fig. 4B). Further analysis showed that the tube length in the c-exo group was 42.90 ± 0.31 mm, while that in the s-exo and PBS groups was only 37.18 ± 0.02 mm and 28.44 ± 0.12 mm, respectively (Fig. 4C). To evaluate the angiogenic effect of exosomes on a molecular level, Q-PCR was performed. Results showed that, compared to the s-exo group, the c-exo group showed a marked upregulation of the pro-angiogenic genes, Flk1 and Ang1, and downregulation of the anti-angiogenic gene TSP1 (Fig. 4D). Hence, our findings suggested that c-exo can directly facilitate tube formation and promote wound healing; however, s-exo has reduced ability to do so.

**Figure 6.**
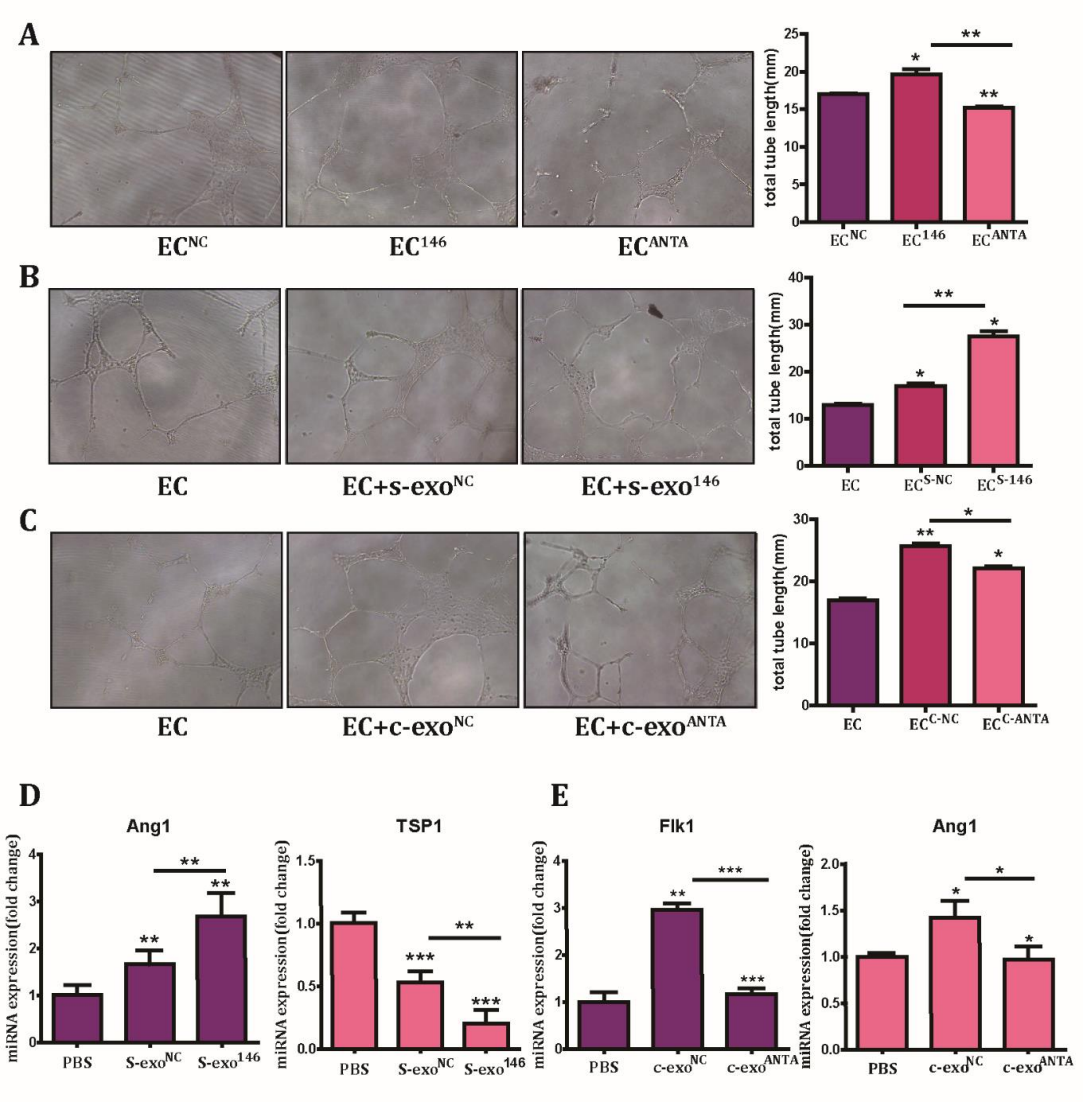
Reduced pro-angiogenic capacity of s-exo was partly mediated by its lower content of miR-146a. (A) The effect of miR-146a overexpression or inhibition on tube formation in HUVECs (EC^146^ or EC^ANTA^) *in vitro*. Scale bar: 100 μm. Quantitative analysis by bar charts (right panel). (B) The tube formation capacity of s-exo transfected with miR-146a (s-exo^146^) or negative control miRNA (s-exo^NC^) in vitro. Scale bar: 100 μm. Quantitative analysis by bar charts (right panel). (C) The tube formation capacity of c-exo transfected with antagomir-146a (c-exo^ANTA^) or negative control miRNA (c-exo^NC^) *in vitro*. Scale bar: 100 μm. Quantitative analysis by bar charts (right panel). (D) Quantification of differences in angiogenesis-associated genes between exosomes and their transfected counterparts.

### Senescence induces changes in expression of exosomal miRNAs, including angiogenesis-associated miR-146a

After confirming that both exosome groups had different angiogenic abilities, we next wanted to identify the specific molecule involved in wound recovery. Since several exosomal miRNAs have been reported to play a crucial role in angiogenesis, we aimed to identify the functional miRNAs associated with the angiogenic effect of exosomes. Exosomal miRNA expression profiles were evaluated. Nine hundred and twenty-six miRNAs were identified in the two types of exosomes (Fig. 5A). Statistical analysis revealed 31 upregulated and 22 downregulated miRNAs in c-exo compared to s-exo (Fig. 5B), and the top 10 up- and down-regulated miRNAs are shown in Figure 5C. The angiogenesis-related miRNAs were then selected and ranked by fold change. However, no significant differences in miRNA expression were found between c-exo and s-exo. We screened origin data of miRNAs expressive abundance and discovered some significant changes were induced only because of low expressive abundance. Further analysis demonstrated that most miRNAs found in exosomes were present in both c-exo and s-exo, suggesting the intersection of miRNA expression profiles. We selected the top 10 miRNAs with the highest expression and a fold change of 1.5 times or more in the intersection and found that miR-146a was the only proangiogenic miRNA (Fig. 5D). Q-PCR quantification results showed that the expression level of miR-146a in c-exo was significantly higher than that in s-exo. Moreover, the expression of other angiogenesis-related miRNAs selected based on fold-change was not significantly altered (Fig. 5E). Hence, these data showed that exosomes are enriched in miRNAs, but their expression profile is markedly altered when the parental cells are senescent. After careful evaluation, we selected miR-146a as a functional miRNA candidate.

### Low miR-146a expression in s-exo partially contributes to its reduced pro-angiogenic capacity

Since we showed earlier that upregulation of miR-146a in HUVECs facilitated tube formation and that miR-146a expression was downregulated in s-exo, we wanted to determine whether the reduced wound healing-promoting effect of s-exo was due to changes in miR-146a expression. For this, HUVECs were transfected with miR-146a, antagomir-146a, or negative control miRNA to perform the tube formation experiment. The results confirmed that increased expression of miR-146a enhanced angiogenesis *in vitro*, while inhibition of miR-146a expression suppressed tube formation (Fig. 6A). To confirm miR-146a function in exosomes, s-exo were transfected with negative control miRNA or miR-146a to detect the effect on tube formation after co-culture for 24 h. Photographs revealed that transfection of s-exo with miR-146a partly recovered its impaired pro-angiogenic capacity (Fig. 6B). Next, c-exo was transfected with antagomir-146a or negative control miRNA and tube formation was evaluated. In keeping with previous experiments, c-exo transfected with antagomir-146a displayed a reduced capacity for promoting tube formation (Fig. 6C). To further verify the effect of exosomal miR-146a on angiogenesis, the expression of angiogenesis-related genes was detected by Q-PCR. Quantification results showed that the pro-angiogenic gene Ang1 was upregulated, while the anti-angiogenic gene TSP1 was downregulated in s-exo transfected with miR-146a compared with that in s-exo transfected with negative control miRNA. In addition, we also found that the expression of the pro-angiogenic genes, Ang1 and Flk1, decreased in c-exo transfected with antagomir-146a (Fig. 6D). Taken together, these results demonstrated that the reduced pro-angiogenic capacity of s-exo was partially due to decreased miR-146a levels.

## DISCUSSION

Due to the self-renewing ability of skin cells, a skin wound usually heals by itself. However, spontaneous regeneration is deficient in cases of full-thickness wounds caused by acute injury or pathological disorders. Numerous therapies are available for full-thickness wounds, including skin grafts, stem cell transplantation, and exosome treatment [29]. Emerging evidence suggests that exosome treatment has increasing potential for wound healing because proteins inside or on the surface of exosomes possess catalytic activity, which may reduce the risk of over-or under-dosing [30]. Moreover, lower immunogenicity and higher safety make it a better replacement for cell-based therapy and a promising method for clinical use. To isolate exosomes of good quality, the isolation method, parental cell state, and storage conditions must be optimized. Although time-consuming, ultracentrifugation is the golden standard for isolating exosomes [31]. It is well-known that exosomes should be kept in -80°C to preserve their bioactivity [32]. As for parental cell state, which is a neglected factor, emerging evidence suggests that it has a key impact on exosome content and function. Eitan et al. showed that lysosome status affects extracellular vesicle content and release [33]. Tomohiro Umezu et al. demonstrated that exosomes derived from older bone marrow stromal cells have reduced function and can be replenished through miR-340 transfection [34]. MSC-derived exosomes have been reported to have low immunogenicity because of similar composition to parental cells [35]. Therefore, to develop a promising exosome-based therapy, understanding how parental cell state, especially senescence, changes the exosome composition and then induces functional alterations is paramount. Here, we used c-exo and s-exo. No significant differences in characterization and secretion were found between the two exosome types. Davis et al. previously demonstrated that extracellular vesicles isolated from young and aging bone marrow stem cells exhibit similar morphology and size distribution [36]. Although they have similar characteristics, c-exo and s-exo possessed different pro-wound healing capacities. s-exo had reduced wound healing ability compared with c-exo. Many studies have reported that MSC-exosomes promote endothelial cell angiogenesis [37, 38]. Taking into account the results of tube formation, we postulated that the reduced wound healing effect of s-exo can be attributed to its impaired pro-angiogenic ability. The *in vivo* matrigel plug assay further showed that the pro-angiogenic effect of s-exo was lesser compared with that of c-exo *in vivo*. The results of *in vitro* tube formation assay were consistent with previous data.

How exosomes cause significant cellular changes in receptor cells requires further research. Using microarray analysis, Lang et al. revealed that MSC-derived exosomes are enriched in miR-124a, which is an effective antiglioma molecule worthy of further clinical evaluation [39]. Li et al. showed that exosomes from bone marrow-derived MSCs increase the population of cancer stem cells (CSCs) via transfer of miR-142-3p [40]. Here, we detected the miRNA expression patterns of the two kinds of exosomes. To identify the candidate miRNA contributing to the reduced pro-angiogenic capacity of s-exo, we selected 31 upregulated miRNAs and 22 downregulated miRNAs in c-exo compared with s-exo, and confirmed miR-146a as the functional miRNA in facilitating angiogenesis. It has been reported that miR-146a negatively modulates the senescence-associated inflammatory mediators, interleukin (IL)-6 and IL-8, in senescent cells [41]. miR-146a has been demonstrated to be correlated with a variety of signaling pathways. As an anti-inflammatory molecule [42], miR-146a was shown to attenuate hepatocyte apoptosis [43] and murine allergic rhinitis [44] by downregulating the TLR4/NF-κB pathway. Additionally, miR-146a has been shown to modulate macrophage polarization by inhibiting the Notch1 pathway [45], and regulate diabetic glomerulopathy in podocytes via the Notch1 pathway [46]. With regard to the proangiogenic effect of miR-146a, Rau CS *et al.* [47] proposed that miR-146a may play a role in regulating angiogenesis in HUVECs by downregulating the expression of CARD10, which acts in a negative feedback regulation loop to inhibit the activation of NF-κB that, in turn, impairs angiogenesis. Su ZF *et al.* [48] showed that miR-146a/b promoted the proliferation, migration, and angiogenic ability of endothelial progenitor cells through downregulation of tumor necrosis factor receptor-associated factor 6 and interleukin-1 receptor-associated kinase 1 expression. Furthermore, Li Y *et al.* [49] found that miR-146a-mediated TGF-β inhibition contributed to angiogenesis. Here, we proposed that the proangiogenic effect of miR-146a may be related to a complex signaling network, and further studies are required to elucidate the exact underlying molecular mechanisms. Our findings showed that miR-146a overexpression had pro-angiogenic effect, and the inhibition of tube formation in antagomir-146a-transfected cells could be rescued by the addition of c-exo and s-exo, thus suggesting that miR-146a modulates exosome function in HUVECs. Further experiments demonstrated that overexpression of miR-146a in s-exo rescued its reduced pro-angiogenic capacity and downregulation of miR-146a in c-exo inhibited its tube formation ability.

In conclusion, we found that exosomes derived from MSCs exhibited pro-angiogenic ability; however, this ability was reduced or even lost when the parental cells were senescent. This decreased ability of s-exo was caused by changes in the expression patterns of miRNAs, especially the reduction of pro-angiogenic miR-146a expression. However, several issues require future investigation, including which surface receptor plays a role in exosome internalization, which target genes modulate exosomal miR-146a function in HUVECs, and whether other exosomal miRNAs play a role in angiogenesis.

## Supplementary Materials

The Supplemenantry data can be found online at: www.aginganddisease.org/EN/10.14336/AD.2020.0624.


