# Supplementary material for: Hydrogen Peroxide-Induced Senescence Reduces the Wound Healing-Promoting Effects of Mesenchymal Stem Cell-Derived Exosomes Partially via miR-146a
Source: Aging Dis. 2021 Feb 1;12(1):102–15. doi: 10.14336/AD.2020.0624 (PMC7801275; doi:10.14336/AD.2020.0624)
Supplement: Supplementary file 1 [file AD-12-1-102-s.pdf]

SUPPLEMENTARY DATA

Hydrogen Peroxide-Induced Senescence Reduces the Wound Healing-Promoting Effects of Mesenchymal Stem Cell-Derived Exosomes Partially via miR-146a

Meiqian Xu[1,#], Xiaodong Su[2,#], Xian Xiao[1], Hongliang Yu[1], Xiaoxia Li[3], Armand Keating[4,5,6], Shihua Wang[1*], Robert Chunhua Zhao[1*]

[1]Institute of Basic Medical Sciences Chinese Academy of Medical Sciences，School of Basic Medicine Peking Union Medical College, Center of Excellence in Tissue Engineering Chinese Academy of Medical Sciences, Beijing 100005, China. [2]Brain Tumor Research Center, Beijing Neurosurgical Institute, Beijing Tiantan Hospital Affiliated to Capital Medical University, Beijing Laboratory of Biomedical Materials, Beijing 100070, China. [3]Department of Genetics and Cell Biology, Basic Medical College, Qingdao University, Qingdao 266071, China. [4]Cell Therapy Translational Research Laboratory, Princess Margaret, Cancer Centre, Toronto, Ontario M5G 2M9, Canada. [5]Institute of Biomaterials and Biomedical Engineering, University of Toronto, Toronto, Ontario M5G2M9, Canada. [6]Institute of Medical Science, University of Toronto, Toronto, Ontario M5G 2M9, Canada.

© 2020. Xu M, et al. Published online at http://www.aginganddisease.org/EN/10.14336/AD.2020.0624

SUPPLEMENTARY DATA

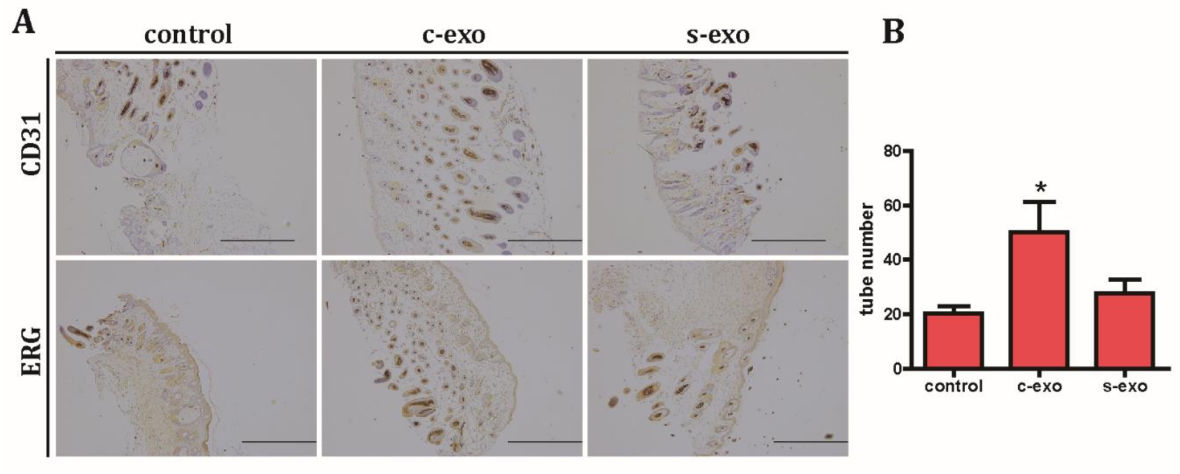

Supplementary Figure 1. Reduced tube numbers around wound in skins treated with s-exo. (A): Immunohistochemical stain with CD31 and ERG in skins. (B): Quantification of positive tube numbers.

© 2020. Xu M, et al. Published online at http://www.aginganddisease.org/EN/10.14336/AD.2020.0624
